# Seed dispersal in *Neuwiedia singapureana*: novel evidence for avian endozoochory in the earliest diverging clade in Orchidaceae

**DOI:** 10.1186/s40529-020-00308-z

**Published:** 2021-01-12

**Authors:** Yu Zhang, Yuan-Yuan Li, Miaomiao Wang, Jia Liu, Fanqiang Luo, Yung-I Lee

**Affiliations:** 1 Beijing Floriculture Engineering Technology Research Centre, Beijing Laboratory of Urban and Rural Ecological Environment, Beijing Botanical Garden, Beijing, 100093 China; 2 College of Plant Protection/Beijing Key Laboratory of Seed Disease Testing and Control, China Agricultural University, Beijing, 100193 China; 3 Administration Bureau of Hainan Diaoluoshan National Nature Reserve, Diaoluoshan Forestry Bureau of Hainan Province, Lingshui County, 572433 Hainan China; 4 Biology Department, National Museum of Natural Science, 40453 Taichung, Taiwan; 5 Department of Life Sciences, National Chung Hsing University, 40227 Taichung, Taiwan

**Keywords:** Apostasioideae, Dispersal, Endozoochory, Germination, Lignification, Seed coat

## Abstract

**Background:**

Seed dispersal allows plants to colonize new habitats that has an significant influence on plant distribution and population dynamics. Orchids produce numerous tiny seeds without endosperm, which are considered to be mainly wind-dispersed. Here, we report avian seed dispersal for an early diverging orchid species, *Neuwiedia singapureana*, which produces fleshy fruits with hard seed coats in the understory of tropical forests.

**Results:**

*Neuwiedia singapureana* produced fleshy fruits that turned red in autumn, and birds were confirmed to be the primary seed dispersers. As compared to its sister species, *N. veratrifolia* with dehiscent capsular fruits, embryos of *N. singapureana* were larger and enclosed by thickened and lignified seed coats. After passing through the digestive tracts of birds, the seeds still stayed alive, and the walls of seed coat contained several cracks. The germination percentage increased significantly for digested seeds as compared with seeds from intact fruits.

**Conclusion:**

The thickened and lignified seed coat may protect seeds as they passed through the digestive tracts of birds. Taken together with a recent report of insect-mediated seed dispersal system in the subfamily Apostasioideae, the animal-mediated seed dispersal may be an adaptive mechanism promoting the success of colonization in dark understory habitats.

## Background

Seed dispersal has an important influence on plant distribution, abundance and population dynamics because it determines the future locations where seeds and later seedlings will survive or expire (Eriksson 2000). In plants, dispersal methods could greatly differ, including by animals (e.g., birds, bats, primates, rodents and fishes), water, wind and gravity (Howe and Smallwood 1982). Various fruit features have been interpreted as coadapted traits of plants that influence the strategy of seed dispersal, such as fruit density, dehiscence, color, palatability, weight and nutrient content (Smith 2001). Orchidaceae is one of the largest angiosperm families with an estimated 800 genera and more than 24,000 species mainly inhabiting subtropical and tropical regions (World Checklist of Monocotyledons, 2006). Upon a successful pollination event in orchids, numerous and tiny seeds are produced in a fruit (Arditti 1992). Furthermore, the tiny orchid seed contains an undifferentiated embryo without endosperm covered by a thin layer of seed coat (Dressler 1993). These characters are considered to be an adaptation for wind dispersal (Arditti and Ghani 2000).

Most orchids possess dehiscent, capsular fruits, while indehiscent, fleshy fruits have been observed only in a few genera across different subfamilies, such as *Neuwiedia* (subfamily Apostasioideae), *Selenipedium* (subfamily Cypripedioideae), *Cyrtosia* (subfamily Vanilloideae), and *Yoania* (subfamily Epidendroideae) (Dressler 1989; Clements and Molvray 1999; Kocyan and Endress 2001; Suetsugu 2018a,b). So far no fleshy fruit is reported in any genus of subfamily Orchidoideae.

The subfamily Apostasioideae (containing two genera—*Apostasia* and *Neuwiedia*) has been considered as the earliest-diverging lineage of Orchidaceae, which possesses several unique characters, including actinomorphic flower, three stamens and powdery pollen grains (Kocyan and Endress 2001). In addition, the presence of indehiscent fruits and the thickened seed coat in some *Apostasia* and *Neuwiedia* species (Nishimura and Tamura 1993; Clements 1999) are suggested to be plesiomorphic characters. Recently, Suetsugu (2020) reported that *Apostasia nipponica* possesses green inconspicuous and indehiscent fruits and depends on cricket/camel cricket species for seed dispersal. In our field investigations, *Neuwiedia singapureana* produces several fleshy fruits on a spike that turn red once they are ripe. This character implies the seed dispersal by birds.

In this study, fruits of *N. singapureana* were monitored by remote cameras to record the consumers. The histological feature of *N. singapureana* mature seeds was investigated. In order to know if the trait of fleshy/capsular fruits is correlated with the seed character, we compared the seed morphology and embryo size of *N. singapureana* and its sister species, *N. veratrifolia* with capsular fruits. To investigate whether the seeds could survive after consumption by birds, we fed the fruits of *N. singapureana* and *N. veratrifolia* to birds and collected seeds from faeces for viability testing by 2,3,5-triphenyl tetrazolium chloride (TTC) staining. The morphology of seed coat between intact and defecated seeds was compared by scanning electron microscope.

## Methods

### Study site and investigations

*Neuwiedia singapureana* is a terrestrial orchid species found in the broad-leaved evergreen forest. Field studies were conducted between 2015 and 2016 in the Diaoluo Mountain region in Hainan, China. Voucher specimens of *N. singapureana* were deposited in the herbarium of the National Museum of Nature and Science, Taiwan (accession nos.: Yung-I Lee 201601). Consumers of *N. singapureana* fruits were monitored in the field by remote cameras that had built-in infrared motion sensors (Acorn Ltl 6210MC Wildlife Camera with 940 nm Covert Infrared & 1080P Video Recording, Zhuhai Ltl Acorn Electronics, Guangdong, China). Each camera was set up 1 m away from 10 *N. singapureana* plants to determine the animals feeding on the fruits. Observations were from 0820 and 1800 h during August 25 to 29 in 2015 and from 0900 to1600 h during August 20 to 23 in 2016.

### Histology

Seeds were fixed in a solution of 2.5% glutaraldehyde in 0.1 M phosphate buffer (pH 6.8) for 24 h at room temperature, then samples were dehydrated with an ethanol series and embedded in Technovit 7100 (Kulzer & Co., Germany) as described (Yeung and Chan 2015). Sections of 3-µm thick were cut with glass knives by using a Reichert-Jung 2040 Autocut rotary microtome, then stained with 0.05% (w/v) toluidine blue O (TBO) in benzoate buffer and examined and captured digitally by using a CCD camera attached to a light microscope (Axio Imager A1, Carl Zeiss AG).

### Comparison of seed morphology

Seed morphology was compared for *N. singapureana* with fleshy fruits and a closely related species, *N. veratrifolia*, with dehiscent, capsular fruits. Voucher specimens of *N. veratrifolia* were deposited in the herbarium of the National Museum of Nature and Science, Taiwan (accession nos.: Yung-I Lee 201232). The mature seeds of both *Neuwiedia* species were collected and dried in a desiccator and the relative humidity was maintained at 30%. The length and width of embryos were measured and recorded in 100 seeds for each species by using a light microscope (Axio Imager A1, Carl Zeiss AG). The embryo volume was calculated according to Arditti and Ghani (2000): [3π × (half length of embryo) × (half width of embryo)^2^/4].

### Seed viability

Mature seeds of *N. singapureana* and *N. veratrifolia* before and after defecation by birds were collected and treated with 0.5% NaOCl solution (w/v) + 0.1% Tween-20 (v/v) for 1 h, then incubated with 1% 2,3,5-triphenyltetrazolium chloride solution at 27 °C for 5 d as described (Lee et al. 2005). Under a dissecting microscope, the embryos remaining yellow were considered unstained (dead), and those turning orange to red were considered stained (viable). The staining tests were replicated 3 times with 100 seeds in each replicate.

### Scanning electron microscopy

For observing the damaged seed coat after consumption by birds, seeds were fixed in 1% (v/v) glutaraldehyde in 0.1 M sodium phosphate buffer, pH 6.8, for 24 h at room temperature. After dehydration in an alcohol–acetone series, samples were critical-point dried, sputter-coated with platinum and observed under a scanning electron microscope (S-4200, Hitachi, Tokyo) with an accelerating voltage of 15 kV.

### Asymbiotic seed germination

To investigate the capacity of seed germination after consumption by birds, we collected 30 fruits that had been consumed by birds and performed asymbiotic seed germination experiments. The seeds defecated by birds were washed and collected for sterilization in 0.5% sodium hypochlorite solution with 0.1% Tween 20 (Sigma-Aldrich) for 15 min. For the control, seeds were sterilized only in sodium hypochlorite solution. After three rinses with sterile distilled water, seeds were placed onto 20-ml modified Murashige and Skoog (MS) medium (Murashige and Skoog 1962) in a 9-cm diameter Petri dish. The modified MS medium contained 1/4 strength macroelements with full-strength microelements (2 mg glycine, 0.5 mg niacin, 0.5 mg pyridoxine HCl, 0.1 mg thiamine, 100 mg myo-inositol, 20 g sucrose and 6 g agar/L). The pH was adjusted to 5.7 before autoclaving at 102 kPa and 121 °C for 20 min. The cultures were maintained in the growth room under darkness at 26 ± 2 °C. Experiments were performed in a randomized design and repeated three times. Twelve plates were used for each treatment, with a minimum of 100 seeds per plate. Each plate was examined and the germination percentage was scored monthly by using a stereomicroscope (Carl Zeiss AG, Germany). Germination was defined as emergence of the embryo from the testa.

### Statistical analyses

The seed viability test and germination experiments were established in a completely randomized design and repeated three times. The data were statistically analyzed by one-way ANOVA followed by Fisher’s protected least significant difference test.

## Results

### Flora and fruit morphology and seed structure

**Fig. 1:**
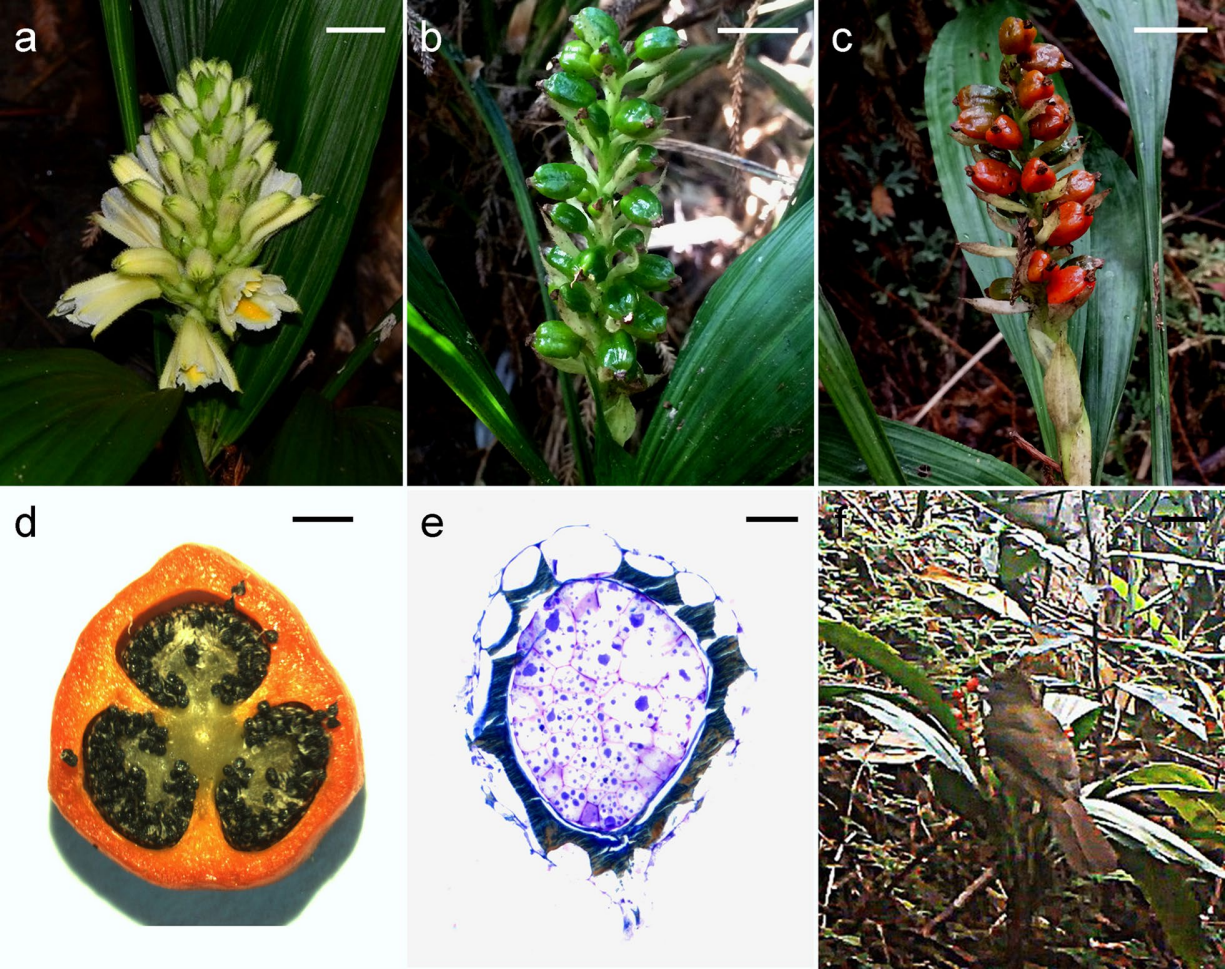
Flowers, developing fruits and seeds of *Neuwiedia singapureana* and its dispersal agents. **a** Flowers.Bar = 1 cm. **b** Immature fruits. Bar = 1 cm. **c** Mature fruits. Bar = 1 cm. **d** Cross section of a mature fruit. Bar = 1 mm. **e** Cross section of a mature seed stained with toluidine blue O. Bar = 60 µm. **f** A bird feeding on the mature fruits. Bar = 3 cm

Each plant produced a single inflorescence bearing multiple creamy-white flowers (Fig. 1a). The flowering period for this species in the Diaoluo Mountain area occurs from June to July, and the ratio of fruit set was relatively high (Fig. 1b). Fruits turned red and became mature in October (Fig. 1c). In the cross section of a mature fruit, three locules were filled with numerous black seeds (mean number of seeds per fruit, 2024.2 ± 390.1) attached to the axile placenta (Fig. 1d). The embryo was about nine cells long and six cells wide (Fig. 1d). In the seed coat, the inner periclinal and anticlinal walls were very thick as compared with the outer periclinal wall (Fig. 1e). Moreover, the thickened wall of the seed coat stained greenish blue with TBO, indicating the presence of phenolic compounds in the walls.

### Fruits consumed by birds

We monitored the fruits of *N. singapureana* for a total of 3,768 h in the field by using motion sensor-equipped cameras: the fruits were commonly eaten by two species of birds (*Alophoixus pallidus* and *Lophura nythemera*) (Table 1; Fig. 1f; Additional file 2: Video S1).

**Table 1:** List of bird species captured by motion sensor camera

Species	2015	2016
*Alophoixus pallidus*	4	26
*Lophura nythemera*	18	12

Camera recordings were conducted for a total of 3,768 h in the field. Numbers given are the total numbers of frames that captured each bird species and individuals that fed on the fruits

### Seed morphometrics

**Fig. 2:**
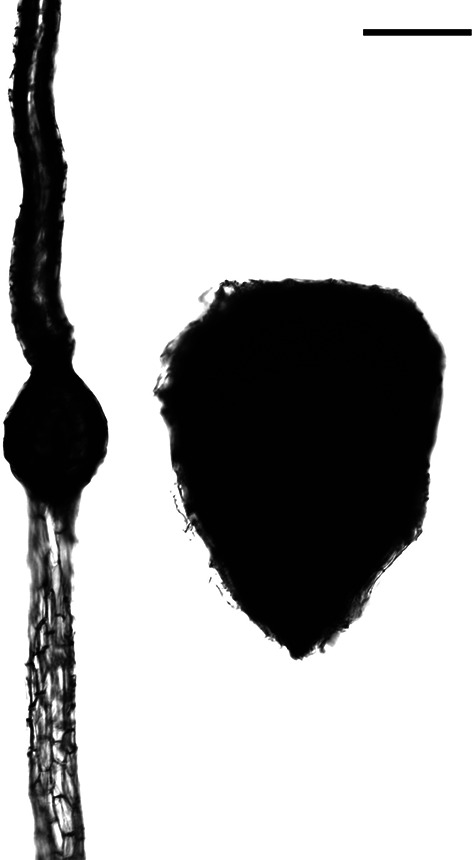
Seed morphology of *N. singapureana* (right) and its sister species, *N. veratrifolia* (left). Bar = 150 µm

*Neuwiedia singapureana* had oval shape seeds within the fleshy fruit, while *N. veratrifolia* had linear seeds within the dehiscent, capsular fruit (Table 2; Fig. 2). Besides, embryo lengths, widths and volumes significantly differed between *N. singapureana* and *N. veratrifolia* (Table 2).

**Table 2:** Seed morphometrics of *Neuwiedia* species

Species	Seed			Embryo		
	Length, µm	Width, µm		Length, µm	Width, µm	Volume, µm^3^
*N. singapureana*	362.72 ± 21.94^b^	303.45 ± 22.53^a^		322.28 ± 25.88^a^	292.14 ± 19.24^a^	8,039,314.84 ± 1,814,669.32^a^
*N. veratrifolia*	3241.67 ± 541.2^a^	96.37 ± 15.21^b^		131.51 ± 18.35^b^	85.25 ± 12.79^b^	298,349.06 ± 115,919.85^b^

Data are mean ± SD. Means within a column followed by the same letter are not significantly different at P = 0.05 by Fisher’s protected LSD test

### Mature seeds and seeds defecated from birds

**Fig. 3:**
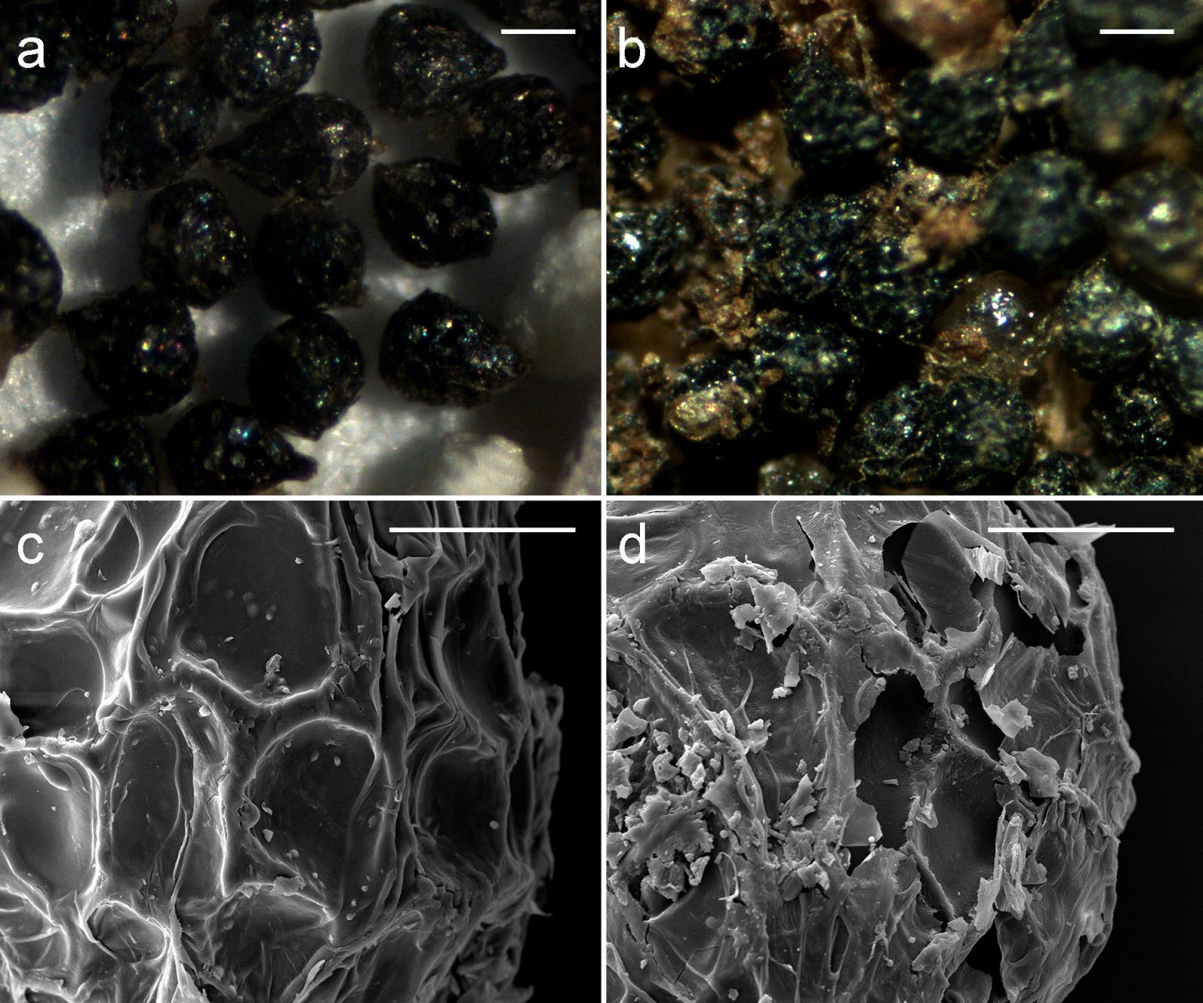
Mature seeds and seeds defecated from birds of *N. singapureana*. **a** Mature seeds. Bar = 300 µm. **b** Seeds defecated from birds. Bar = 300 µm. **c** SEM micrograph showing the surface of mature seed. Bar = 50 µm. **d** SEM micrograph showing the surface of seed defecated from birds. Bar = 50 µm

When mature seeds were collected directly from fruits and air-dried (Fig. 3a), the periclinal wall of the outer layer shrank inwards, which resulted in a concaved surface and reticulated pattern of seed coat (Fig. 3c). For mature seeds from bird faeces (Fig. 3b), the surface of seed coat was eroded, and a number of cracks was observed (Fig. 3d).

### Changes in seed viability and germinability after consumption by birds

**Fig. 4:**
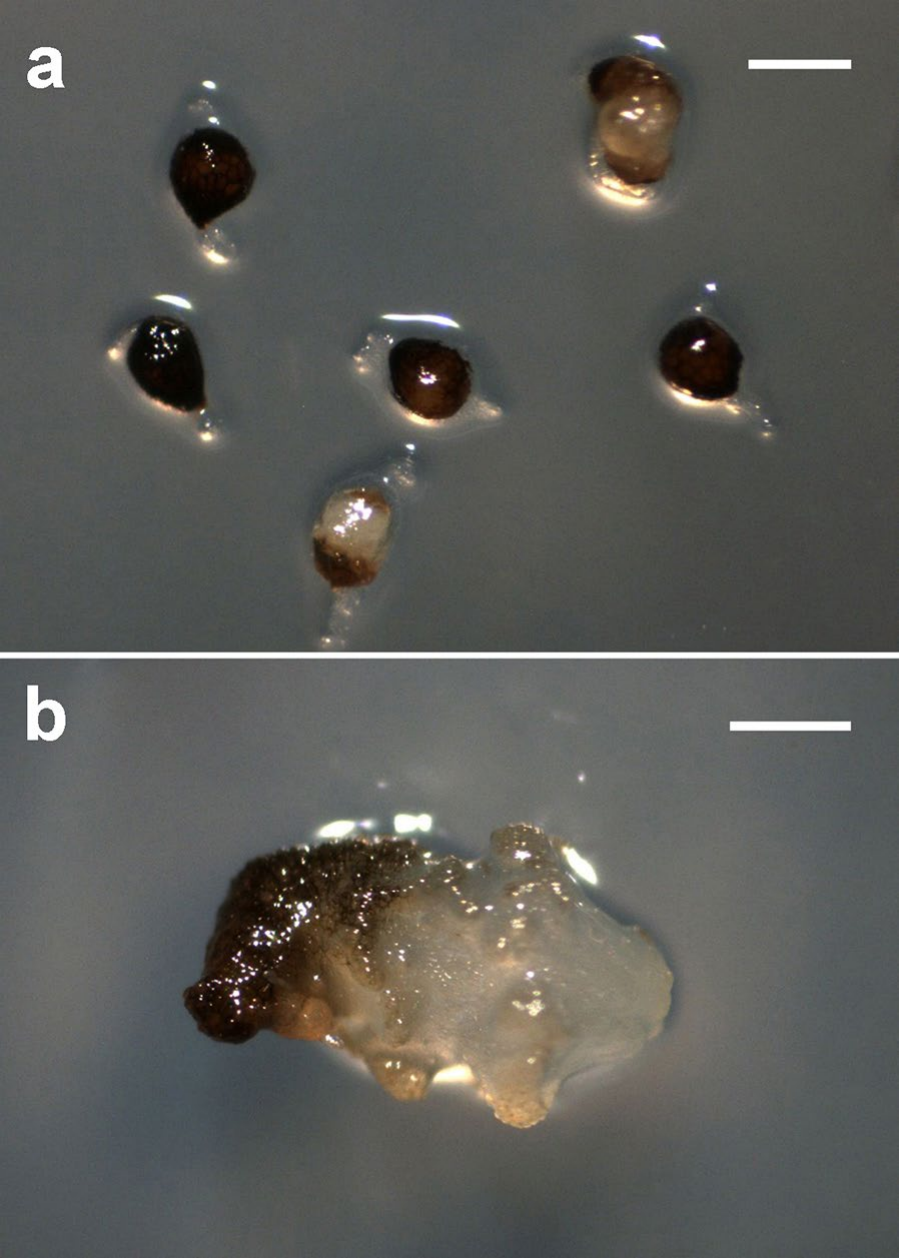
The successive developmental stages of *N. singapureana* from seed germination to protocorm formation in asymbiotic culture in vitro. **a** Light micrograph showing the stages of no growth of embryo and embryos emerging from the seed coat. Bar = 500 µm. **b** Light micrograph showing a developing rhizome-like structure. Bar = 1 mm

To investigate whether the seeds could survive after consumption by birds, we fed the fruits to birds and collected seeds from faeces for viability testing and asymbiotic germination experiments. For the *N. singapureana* seeds collected from faeces, the mean viability was 35.6 ± 4.8% by the TTC stainability test, which was similar to the viability from intact fruits (32.4 ± 8.3%). However, no viable seeds of *N. veratrifolia* were found after consumption by birds (Table 3). Because no seed of *N. veratrifolia* was viable after consumption by birds, we only performed the asymbiotic germination experiments in *N. singapureana* seeds. The seeds collected from faeces had better germination percentage than those from intact fruits (Table 4). By 90 days of culture on 1/4 MS medium, most embryos were still enveloped by the seed coat (Fig. 4a); only a few seeds had become swollen and protruded from the seed coat (Fig. 4a). After germination, the young protocorm enlarged and developed into a small rhizome-like structure (Fig. 4b).

**Table 3:** Effect of feeding treatment on seed viability of *Neuwiedia* species by TTC stainability test

	Seed viability (%)	
	*N. singapureana*	*N. veratrifolia*
Control	32.4 ± 8.3^a^	28.51 ± 7.2^a^
Feeding treatment	35.6 ± 4.8 ^a^	0^b^

Data are mean ± SD. Means within a column followed by the same letter are not significantly different at P = 0.05 by Fisher’s protected LSD test

**Table 4:** Effect of feeding treatment on asymbiotic germination of mature seeds of *Neuwiedia singapureana*

	Days after sowing					
	30	60	90	120	150	180
Control	0^a^	0^a^	0^a^	0^a^	0^a^	0.4 ± 0.1^a^
Feeding treatment	0^a^	0^a^	1.6 ± 0.4^a^	3.2 ± 0.8^b^	5.1 ± 1.6^b^	6.4 ± 1.2^b^

Data are mean ± SD. Means within a column followed by the same letter are not significantly different at P = 0.05 by Fisher’s protected LSD test

## Discussion

Animal-dispersed fleshy fruits are rare in Orchidaceae because most orchid species have capsules that become dry and dehiscent to disperse numerous seeds by wind (Arditti and Ghani 2000). Orchid species with fleshy fruits are primarily observed in the early diverging subfamilies, e.g. *Apostasia* and *Neuwiedia* (subfamily Apostasioideae), *Selenipedium* (subfamily Cypripedioideae), and *Cyrtosia* and *Vanilla* (subfamily Vanilloideae) (Nakamura and Hamada 1978; Dressler 1989; Clements and Molvray 1999). Whereas species with fleshy fruits are not found in the subfamily Orchidoideae, and only *Yoania* is confirmed to produce fleshy fruits in the subfamily Epidendroideae (Suetsugu 2018a,b). Mapping of the traits of fleshy or capsular fruits on a phylogenetic tree based on molecular data (Cameron et al. 1999) suggests that species in the subfamily Apostasioideae have fleshy fruits as a basal character (Additional file 1: Fig. S1). In the subfamily Apostasioideae, only two *Neuwiedia* species, i.e. *N. griffithii* and *N. veratrifolia* are known to possess capsular fruits (Kocyan and Endress 2001). Such an observation would suggest that fleshy fruit might be a plesiomorphic character in Orchidaceae, and the production of fleshy fruits arose a few times in other orchid subfamilies. In monocots and dicots, animal-dispersed fleshy fruits should undergo concerted convergence in association with colonization of a shady environment and have evolved repeatedly (Smith 2001; Givnish et al. 2005). Indeed, up to 95% of the woody understory species in neotropical rain forests bear fleshy fruits (Gentry 1982). Some orchids with fleshy fruits are fully mycoheterotrophic plants; examples are *Cyrtosia* and *Yoania*, inhabiting dark understory habitats with less wind (Suetsugu et al 2015; Suetsugu 2018a,b). Although *N. singapureana* is a green orchid species inhabiting the tropical forest understory, seed dispersal by animals should be an effective way for long-distance dispersal under closed canopies. Recently, *A. nipponica* is found to obtain most of its carbon resource from mycorrhizal association (Suetsugu and Matsubayashi 2020). Partial mycoheterotrophy might be another factor for Apostasioid species to thrive in dark understory habitats. A further study on the seed dispersal systems of five subfamilies using phylogenetic reconstruction of ancestral character states would provide insights into the evolution of seed dispersal system in Orchidaceae.

In this study, birds were confirmed to be the primary dispersers of *N. singapureana* seeds in the understory of tropical forests in China (Fig. 1f; Additional file 2: Video S1). So far, only a fully mycoheterotrophic orchid, *Cyrtosia septentrionalis* was reported to adapt avian endozoochory in the understory of temperate forests in Japan (Suetsugu et al. 2015). Both orchid species have fleshy fruits that turn red in autumn. It has been reported that birds may use color to discover and identify fruits (Wheelwright and Janson 1985). The most conspicuous colors of fleshy fruit to attract birds are red and black, and nearly all birds avoid green fruits (Duan et al. 2014). In *Apostasia* and *Yoania*, their fruits eaten by crickets and camel crickets display green and pinkish white respectively (Suetsugu 2018b, 2020). In *N. singapureana* and *C. septentrionalis*, the selection by birds for increased fruit attractiveness may raise investment in energy in producing the color display, such as carotenoid biosynthesis. It is also notable that fleshy fruits of *Apostasia* and *Yoania* at the ground level have to associated with endozoochory by terrestrial invertebrates (2018a, b and 2020). While both *C. septentrionalis* (Suetsugu et al. 2015) and *N. singapureana* possess the robust stem as a perch that facilitates fleshy fruit consumption by birds. Within the subfamily Apotasioideae, the occurrence of different strength and length of stem types may be a morphological adaptation to dissimilar seed disperser.

It is noteworthy that the indehiscent, fleshy fruit is regularly accompanied with hard, crustose seed coats in Orchidaceae (Nishimura and Tamura 1993; Clements and Molvray 1999; Yang and Lee 2014). In this study, greenish-blue staining with TBO in the thickened wall of the seed coat of *N. singapureana* indicated the accumulation of lignin in the coat wall. Furthermore, the lignified seed coat was much thicker for *N. singapureana* than *N. veratrifolia* (Fig. 2), the wind-dispersed sister species. The thickened lignified seed coat also occurred in orchids with fleshy fruits, such as *Cyrtosia* (Yang and Lee 2014; Suetsugu et al. 2015) and *Yoania* (Suetsugu 2018a,b), which suggests an adaptive trait of seed development. After passing through the digestive tracts of birds, most seeds of *N. singapureana* remained alive. However, no viable seeds of *N. veratrifolia* were detected after passing through the digestive tracts of birds (Table 3). The heavily lignified seed coat may protect the seeds as they pass through the digestive tracts of birds.

Seeds of *N. singapureana* are ellipsoid to ovoid in shape rather than the linear seeds in the dehiscent capsule of its sister species *N. veratrifolia* (Barsberg et al. 2018). Furthermore, the embryo is much larger for *N. singapureana* than its sister species *N. veratrifolia* (Fig. 2). Such enlarged embryo in Orchidaceae was reported for seeds of fleshy fruits (i.e., *Cyrtosia* [Yang and Lee 2014] and *Yoania* [Suetsugu 2018b]). In orchids, the seed morphology, or embryo size, may be related to the different lifestyles. In *Liparis*, the larger embryo of epiphytic *L. fujisanensis* has more cells than the smaller embryo of terrestrial *L. koreana* and *L. kumokiri*, but their cell size does not considerably differ (Tsutsumi et al. 2007). Moreover, the embryo cells are larger for *N. singapureana* than its sister species *N. veratrifolia*. Whether the morphological traits of seeds are related to the evolution of fleshy fruits remains to be analysed.

The feeding treatment resulted in several cracks and removed the wall covering on the seed coat of orchids (Fig. 3). The germination percentage increased significantly after the feeding treatment as compared with seeds from intact fruits (Table 4), which suggests that the dormancy was broken by the digestive tract. In *Yoania japonica*, higher germination was observed in seeds defecated by camel crickets than in seeds collected directly from fruits (Suetsugu 2018b). Thick seed coats of many species are responsible for restricting the diffusion of water, nutrients and oxygen to the embryo (Simpson 1990; Corbineau and Come 1995). Passage through digestive systems of animals can scarify the seed coat and thus improve germination, especially for seeds with a hard seed coat (Howe and Smallwood 1982). Similar mechanisms may also be involved in this orchid dispersal system. The increase in the exchange of materials across the seed coat may lead to the release of dormancy. Thus, under the natural condition, seed dispersal by birds may have facilitated the subsequent germination and colonization of *N. singapureana*.

## Conclusions

In this study, birds were the primary dispersers of *N. singapureana* seeds. As the fruits matured, they turned from green to red, then the birds started to eat the red fruits. After passing through the digestive tracts of birds, the seeds still stay alive, and the walls of the seed coat contain several cracks. The germination percentage increased significantly for digested seeds as compared with intact fruit seeds. The thickened and lignified seed coat may protect seeds as they pass through the digestive tracts of birds. The animal-mediated seed dispersal strategy may be an adaptive mechanism promoting the success of colonization in dark understory habitats.

## Supplementary Information

**Additional file 1: Figure S1.** The occurrence of fleshy fruit in orchid genera mapped onto a phylogenetic framework based on results published by Cameron et al. (1999).

**Additional file 2: Video S1.** Birds eating mature fruits of *Neuwiedia singapureana*.

## Data Availability

Not applicable.
