# Supplementary figures and images for: Seed dispersal in Neuwiedia singapureana: novel evidence for avian endozoochory in the earliest diverging clade in Orchidaceae
Source: Bot Stud. 2021 Jan 12;62:3. doi: 10.1186/s40529-020-00308-z (PMC7803844; doi:10.1186/s40529-020-00308-z)

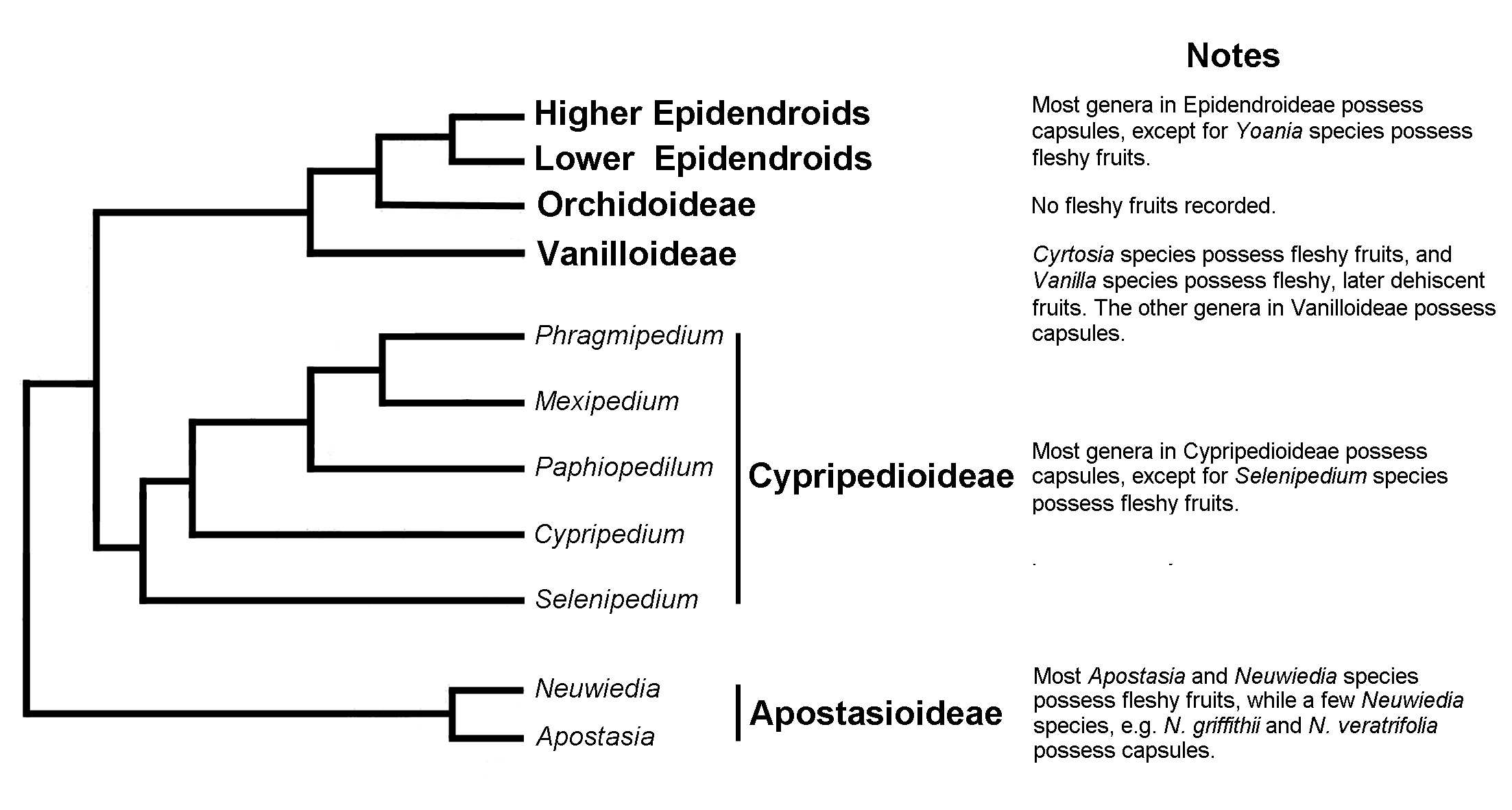

Supplement: Supplementary file 1 — Additional file 1: Figure S1. The occurrence of fleshy fruit in orchid genera mapped onto a phylogenetic framework based on results published by Cameron et al. (1999). [file 40529_2020_308_MOESM1_ESM.jpg]
